# Production and evaluation of parathyroid hormone receptor_1_ ligands with intrinsic or assembled peroxidase domains

**DOI:** 10.1038/s41598-017-13548-0

**Published:** 2017-10-12

**Authors:** Xavier Charest-Morin, Patrice E. Poubelle, François Marceau

**Affiliations:** Division of Infectious Disease and Immunity, CHU de Québec-Université Laval, Quebec City, QC G1V 4G2 Canada

## Abstract

Parathyroid hormone (PTH) can be C-terminally extended without significant affinity loss for the PTH_1_ receptor (PTHR_1_). We developed fusion protein ligands with enzymatic activity to probe PTHR_1_s at the cell surface. Two fusion proteins were generated by linking PTH to the N-terminus of either horseradish peroxidase (PTH-HRP) or the genetically modified soybean peroxidase APEX2 (PTH-APEX2). Alternatively, myc-tagged PTH (PTH-myc) was combined with antibodies, some of which HRP-conjugated, in the extracellular fluid. The three PTH-fusion proteins were produced as conditioned mediums (CM) by transfected producer HEK 293a cells. Binding of receptor-bound enzymatic ligands was revealed using widely available substrate/co-substrate systems. The stimulation of recipient HEK 293a expressing PTHR_1_s with the PTH-myc/antibodies combination or with PTH-APEX2 supported the histochemical or luminescent detection of recombinant PTHR_1_s (TrueBlue^TM^ or luminol-based reagent). The PTH-HRP construction was the most sensitive and supported all tested peroxidase co-substrates (TrueBlue^TM^, tetramethylbenzidine (TMB), luminol, biotin-phenol with streptavidin-Qdots); the 3 latter schemes identified endogenous PTHR_1_ in the osteoblastic HOS cell line. The specificity of the fusion protein binding to PTHR_1_ was determined by its competition with an excess of PTH_1–34_. Bifunctional ligands possessing enzymatic activity detect intact receptors with various possible applications, including the screening of drugs that compete for receptor binding.

## Introduction

Parathyroid hormone (PTH), is an important endocrine mediator involved in the regulation of calcium and phosphate concentrations^1^. Indeed, PTH is an 84 amino acids peptide that is being used clinically, along with the shorter fragment PTH_1–34_ (teriparatide), for the treatment of osteoporosis^2^. The intermittent administration of PTH is used to stimulate bone formation in these patients through the action of this hormone on the osteoblasts via the PTH_1_ receptor (PTHR_1_). PTH is secreted from the parathyroid gland in response to the lowering of blood Ca^2+^ concentrations and will increase Ca^2+^ concentrations by stimulating the PTHR_1_^3,4^. This receptor binds parathyroid hormone-related protein (PTHrP) as well. The sequence of PTHR_1_ is 593 residues long and it is a member of the G-protein coupled receptor (GPCR) B family (secretin family). Like all receptors from this group, the PTHR_1_ possesses large C-terminal and N-terminal domains^5^. Following agonist stimulation, this receptor is rapidly desensitised through phosphorylation of its C-terminal domain via the activity of protein kinase A, protein kinase C or some G-protein coupled receptor kinases (GRKs)^6,7^. The phosphorylation of the C-terminal domain is crucial for the agonist stimulated endocytosis of the PTHR_1_^8^. Following its internalisation, through a clathrin-dependant mechanism, the receptor will progress into the endosomal system leading to the recycling or degradation of the internalised PTHR_1_^9^.

Class B GPCRs are organised in two domains: one extracellular domain involved in the affinity and specificity of ligand binding and a transmembrane domain required for the activation of the receptor^10^. This two domain model suggests that the N-terminal domain of PTH will interact with the extracellular domain of the receptor whereas the C-terminal domain of the hormone will interact with the transmembrane domain. A shorter version of intact PTH, PTH_1–34_ (teriparatide), can stimulate the PTHR_1_ with equivalent affinity (K_I_ of 4 nM vs. 2 nM, respectively, in a radioligand competition assay)^11^. Moreover, the crystal structure of agonist-bound PTHR_1_ extracellular domain showed that the C-terminal domain of the hormone does not bind directly with the receptor^12^. This led to the prediction that PTH could be prolonged at its C-terminal terminus to construct bifunctional receptor ligands.

A fusion protein made of the bright fluorescent protein enhanced green fluorescent protein (EGFP) fused to the C-terminal of PTH_1–34_ yielding PTH_1–34_-EGFP was recently reported^13^. The resulting fusion protein labeled recombinant PTHR_1_s in transfected HEK 293a cells either in microscopy or in flow cytometry, while failing to image receptor populations in an osteoblastic cell line. We also characterised a fusion protein consisting of two antigenic tags fused in tandem to the C-terminal of intact PTH. The two tags were the FLAG tag (DYKDDDDK) and the myc tag (EQKLISEEDL) and the resulting fusion protein was termed PTH-myc^14^. This construction, complexed in the extracellular fluid with AlexaFluor488-conjugated anti-myc antibodies, supported the study of PTHR_1_ cycling. Another biotechnological form of ligand was previously reported where the PTH_1–34_ based agonist was elongated at its C-terminus with a linker and a transmembrane tether^15,16^.

The objective of the current work is to develop new PTH-conjugated fusion proteins that will allow the detection of endogenous population of PTHR_1_s in a largely species-independent manner. To achieve this objective, we fused intact PTH to the N-terminal of an enzyme hoping that the reaction catalysed by this enzyme will support signal amplification to detect cell surface receptors. Since intact GPCRs are difficult to detect using antibodies, especially in living cells, we believed that this approach could be promising and generalizable since all class B GPCRs bind their receptor in the same fashion as PTH.

## Results

### Expression of the fusion proteins

**Figure 1:**
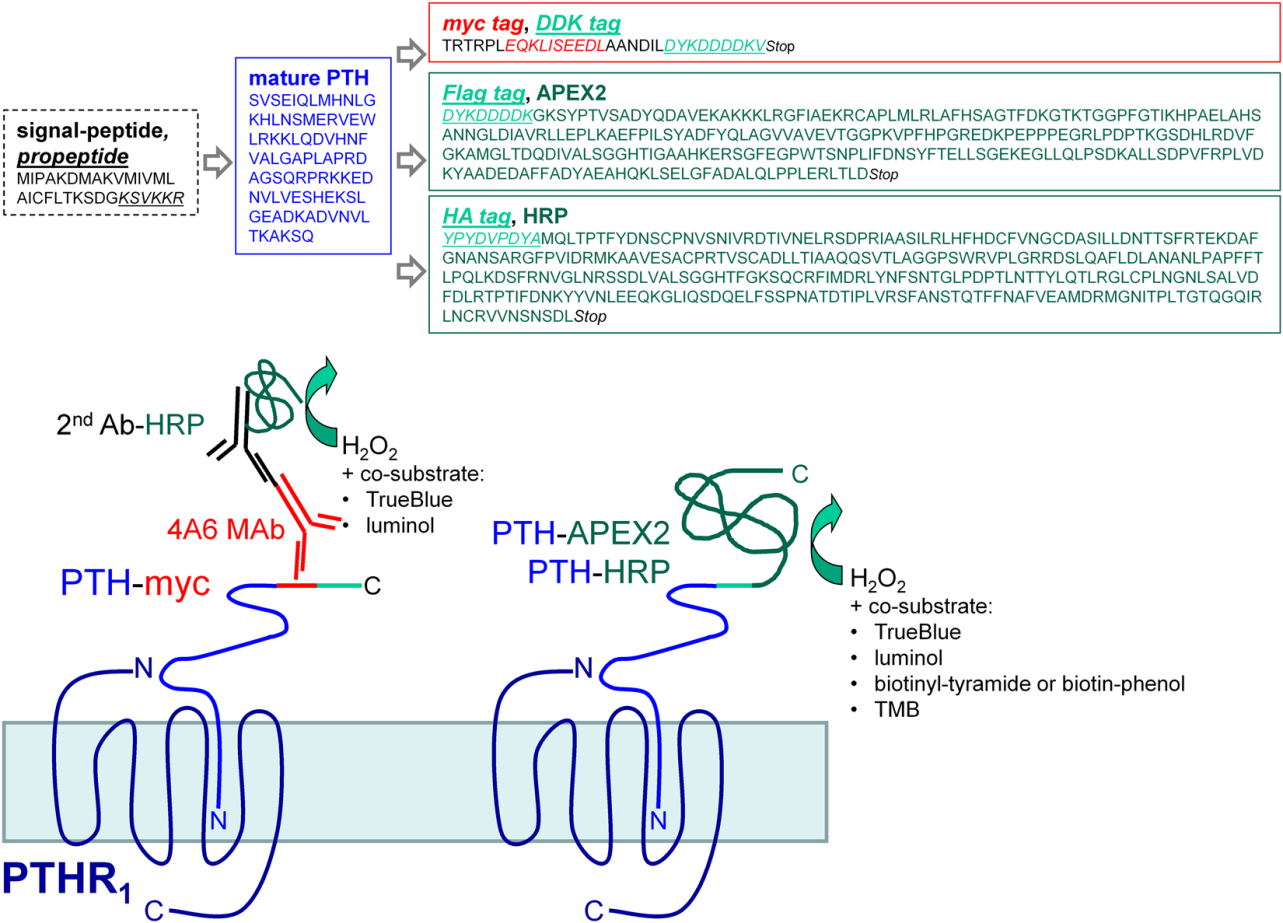
Schematic representation of the design of fusion proteins evaluated as potential PTHR_1_ ligands. The fusion proteins evaluated in this paper consist of the full sequence of prepro-PTH_1–84_ fused to the N-terminus of either a peroxidase (PTH-APEX2 or PTH-HRP) or an antigenic domain containing two epitopes (PTH-myc) recognized by commercially available monoclonal antibodies. The peroxidase domains, either covalently bound to PTH or assembled through antibodies, were detected using either a colorimetric substrate (TrueBlue™ or TMB), a luminescent substrate (luminol) or, alternatively, biotin-phenol or biotinyl-tyramide, peroxidase substrates whose oxidized states form covalent bonds leading to a proximal accumulation of biotin which can be detected by conjugated streptavidin molecules.

**Figure 2:**
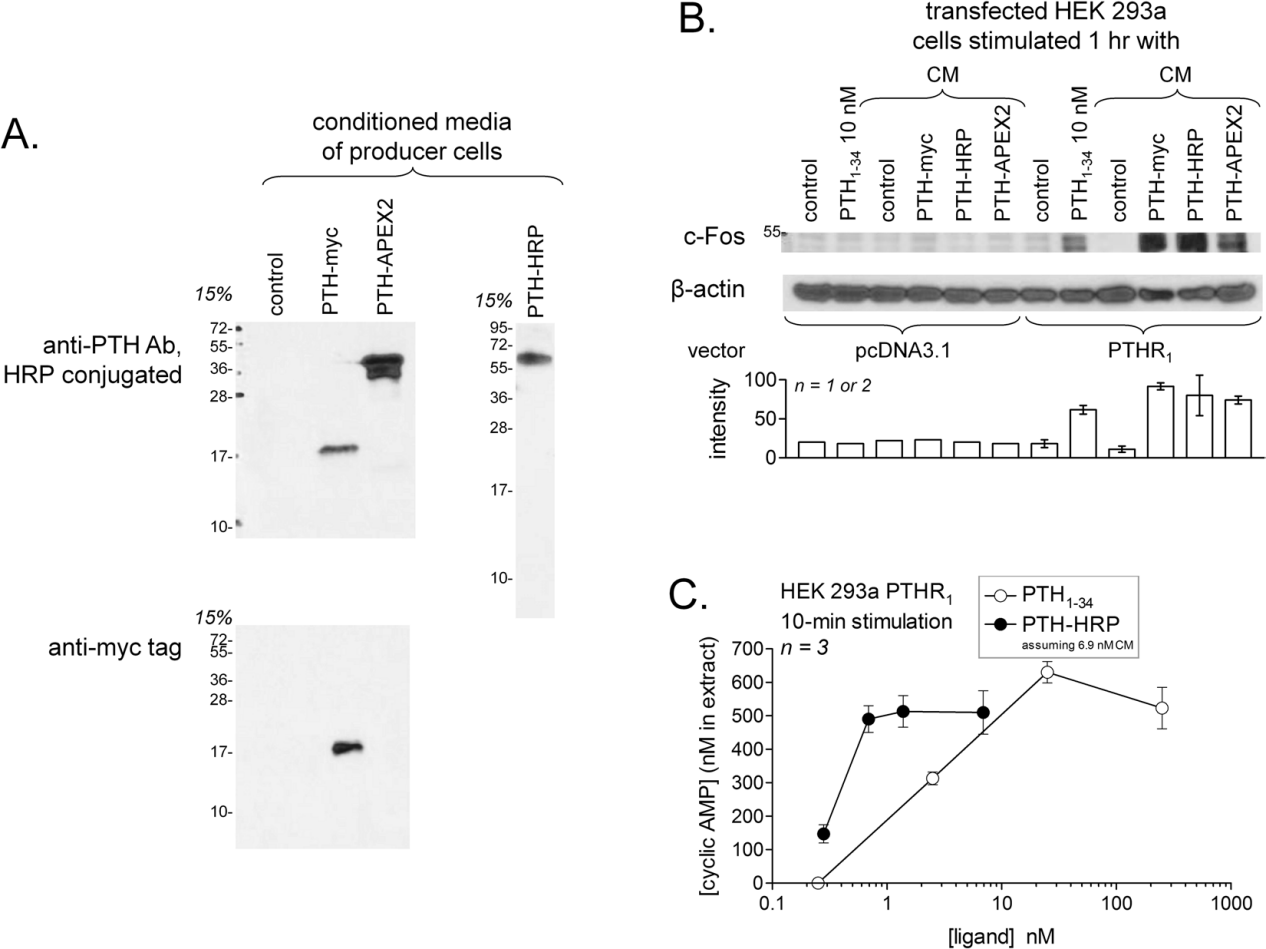
Characterization of the PTH fusion proteins. (**A**) Immunoblots of the CMs of HEK 293a producer cells transiently expressing either pcDNA3.1, PTH-myc, PTH-APEX2 or PTH-HRP. The CMs were loaded on a 15% polyacrylamide gel and were later detected using either an anti-PTH antibody or an anti-myc antibody. (**B**) Signaling (c-Fos accumulation) induced by the 3 fusion proteins and PTH_1–34_ in recipient HEK 293a cells that express PTHR_1_ or pcDNA3.1. (**C**) Cyclic AMP production in petri dishes of HEK 293a transiently expressing PTHR_1_ and acutely (10-min) stimulated as indicated. Cyclic AMP levels were below the limit of detection (~0.5 nM) in cells stimulated with either the saline vehicle of PTH_1–34_ or the undiluted CM of mock-transfected cells. Results are means ± s.e.m. of triplicate determinations.

The three fusion proteins (PTH-APEX2, PTH-HRP and PTH-myc; predicted sequences in Fig. 1) were all produced as conditioned mediums (CMs). To validate the expression and the correct secretion of these proteins, an immunoblot was performed on the CM from each transiently transfected producer cells. The PTH-based fusion proteins were all secreted in the culture medium of these cells consistent with the presence of the prepro-sequence of PTH in each vector. Each fusion proteins had a migration consistent with the molecular weight predicted according to their nucleotide sequence (PTH-APEX2: 38.7 kDa, PTH-HRP: 44.9 kDa and PTH-myc: 13.1 kDa; Fig. 2A). The proteins were detected as single bands, except for PTH-APEX2 which may contain minor degradation products along with the predicted protein. The presence of the PTH domain of each protein, crucial for receptor binding, was validated by the reaction with the anti-PTH antibody; PTH-myc reacted with an anti-myc tag antibody as well (Fig. 2A). The concentration of the PTH-based fusion proteins in CMs of producer HEK 293a cells was estimated using an ELISA for intact PTH in the same pooled CMs used in Fig. 2: the values were PTH-APEX2: 6.1 nM; PTH-HRP: 6.9 nM and PTH-myc 35 nM (control CM contained < 0.1 nM). We previously have shown that PTH-myc elicited PTHR_1_-mediated signaling^14^. This was confirmed and extended to PTH-APEX2 and PTH-HRP: all 3 constructions, as well as synthetic PTH_1–34_, induced c-Fos expression in HEK 293a cells that expressed PTHR_1_, but not in mock-transfected cells (immunoblots, Fig. 2B). Based on cyclic AMP generation in HEK 293a cells that expressed PTHR_1_, we previously determined that PTH-myc was slightly less potent (~5-fold) than synthetic PTH to generate the second messenger cyclic AMP^14^. Using a similar approach, PTH_1–34_ has the same threshold concentration, 2.5 nM, as the full PTH sequence to acutely increase cyclic AMP in receptor expressing cells (Fig. 2C)^14^. However, the most interesting novel fusion protein, PTH-HRP, is about 5 fold more potent than PTH_1–34_ in this assay (Fig. 2C). A possible limitation of these estimates is that the concentration of the fusion proteins in CMs is determined by a PTH ELISA that may be influenced by the extended flanking sequences.

### Detection of PTHR_1_ with PTH-myc using an assembled peroxidase domain

**Figure 3:**
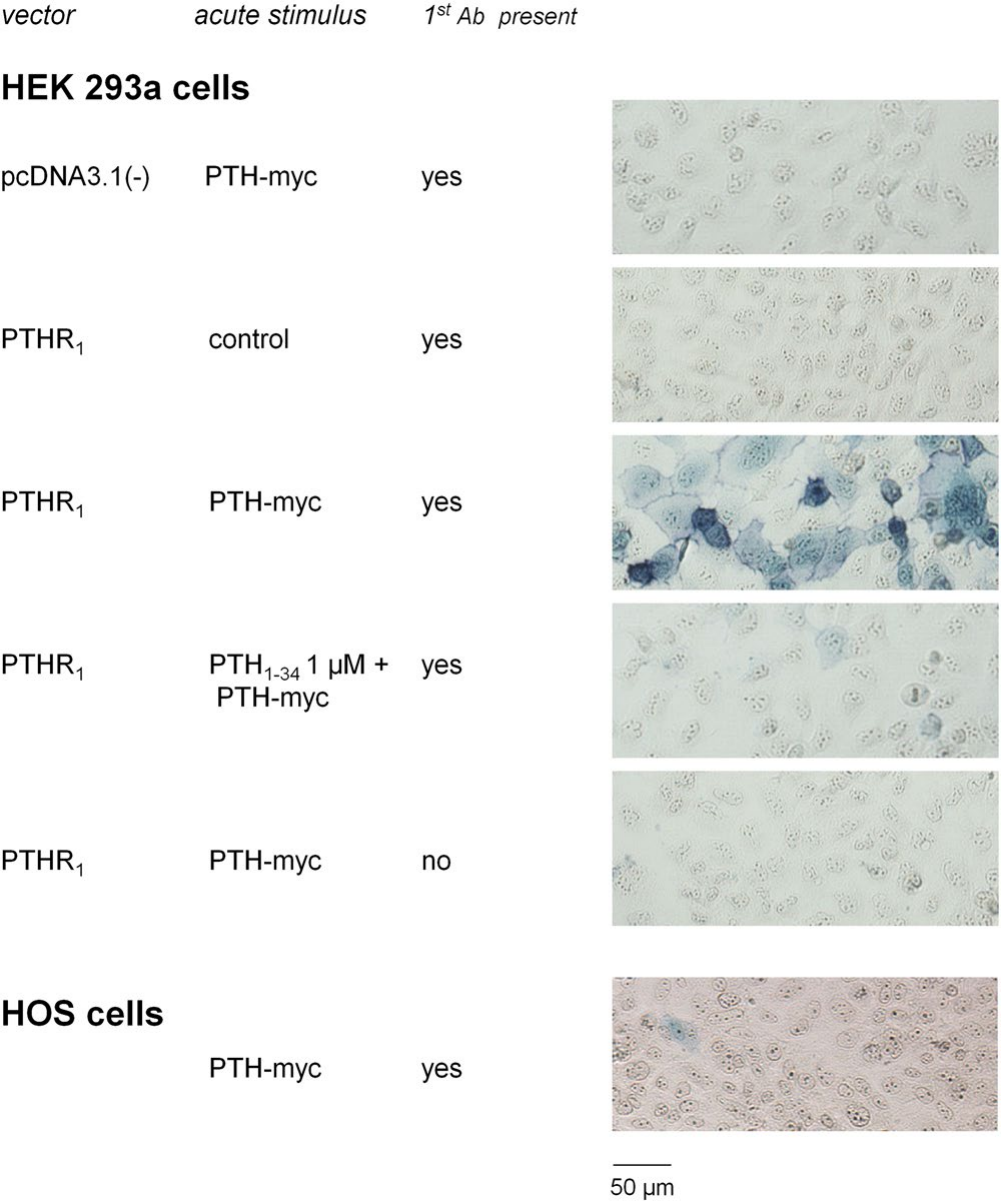
TrueBlue™ labeling of intact cells following stimulation with PTH-myc. HEK 293a transfected cells (PTHR_1_ or pcDNA3.1) or an osteoblastic cell lines (HOS) were stimulated with PTH-myc or control CM. The CMs used in this experiment were supplemented with mouse monoclonal anti-myc antibody (clone 4A6; final concentration 7 nM) and goat anti-mouse IgG antibody conjugated to HRP (final concentration 5.6 nM). The cells were treated for 30 minutes at 37 °C. Following this incubation the cells were rinsed three times before being incubated 10 minutes at room temperature with the peroxidase substrate TrueBlue™. Then, the cells were observed in transmission (final magnification 100×).

Recipient HEK 293a cells transiently transfected with PTHR_1_ or pcDNA3.1 were simultaneously treated with either PTH-myc CM or control CM and with an anti-myc antibody (clone 4A6) plus a secondary antibody coupled to HRP (Fig. 3, histochemistry). The trimolecular complex PTH-myc/4A6/HRP-conjugated secondary antibody successfully labeled HEK 293a cells expressing PTHR_1_ while there was no labeling of cells transfected with empty pcDNA3.1, supporting the specificity of PTH-myc. Moreover, co-treatment of the PTHR_1_-expressing cells with PTH_1–34_ 1 µM, an excess of untagged ligand, largely abrogated the labeling of these cells by the trimolecular complex. Also, cell stimulation with PTH-myc and the secondary antibody conjugated to HRP in the absence of the primary anti-myc antibody failed to support labeling, confirming the specificity of the procedure. However, this system failed to label the endogenous PTHR_1_s population in HOS cells (Fig. 3).

**Figure 4:**
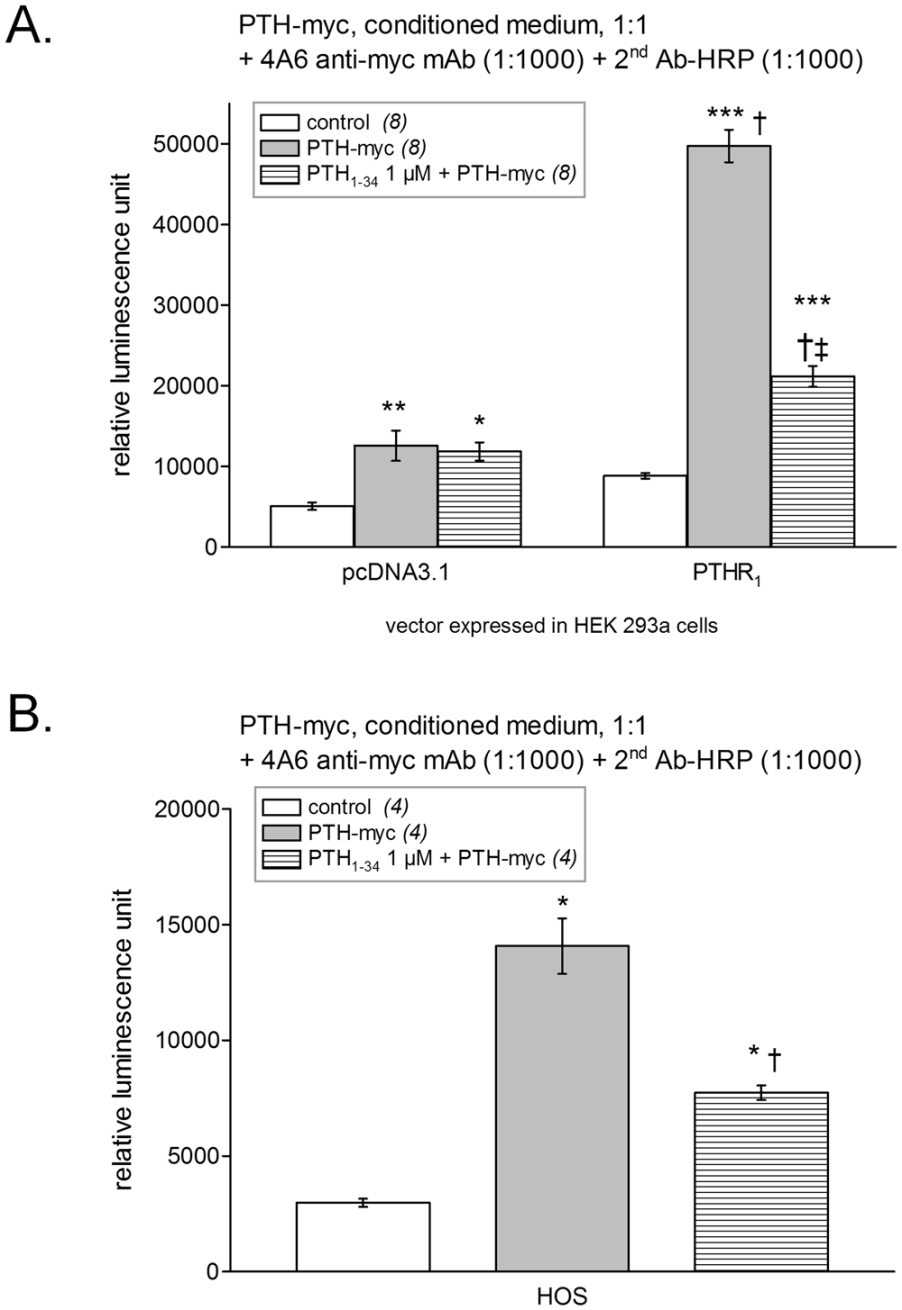
Luminescent detection of PTHR_1_s using the ligand PTH-myc revealed using anti-myc and secondary HRP-conjugated antibodies. (**A**) HEK 293a cells transfected with either an empty vector or one encoding for PTHR_1_. The number of replicates is indicated between parentheses. ANOVA indicated that values were heterogenous (P < 10^−4^). Tukey’s multiple comparison test was applied to compare pairs of values. For each vector, values vs. their respective controls: ^*^P < 0.05; ^**^P < 0.01; effect of adding an excess of PTH_1–34_ vs. the enzymatic ligand alone: ^‡^P < 0.001. Comparison of each experimental condition between vectors: ^†^P < 0.001. (**B**) Endogenous receptors detected in HOS cells. ANOVA indicated that values were heterogenous (P < 10^−4^). Each treatment vs. control value: *P < 0.001; effect of adding an excess of PTH_1–34_ vs. the enzymatic ligand alone: ^†^P < 0.001 (Tukey’s multiple comparison test).

The sensitivity of histochemistry detection of PTHR_1_ was compared to that of a luminol-based luminescent assay as described in Methods. When the latter assay was applied to transfected cells in 24-well plates, a strong luminescent signal was detected from HEK 293a cells expressing PTHR_1_ and treated with PTH-myc (P < 0.001 vs. control receptor-expressing cells). This signal was significantly reduced by a co-stimulation with PTH_1–34_ 1 µM (P < 0.001, Fig. 4A). Mock transfected cells treated with PTH-myc and antibodies were associated with a small but significant luminescence (P < 0.01) that was not abated by co-incubation with PTH_1–34_, indicating some non-specific binding of the trimolecular complex under the applied conditions. To evaluate the capacity of PTH-myc to detect naturally expressed levels of PTHR_1_s, we applied the same protocol to HOS cells (Fig. 4B). The trimolecular complex formed of PTH-myc, an anti-myc antibody and a secondary antibody conjugated to HRP showed significant binding to HOS cells (P < 0.001 vs. control), a fraction of which corresponding to PTHR_1_s as supported by the significant reduction in luminescent labeling produced by a co-treatment with PTH_1–34_ 1 µM (P < 0.001).

### Labeling of PTHR_1_ expressing cells with PTH-APEX2

**Figure 5:**
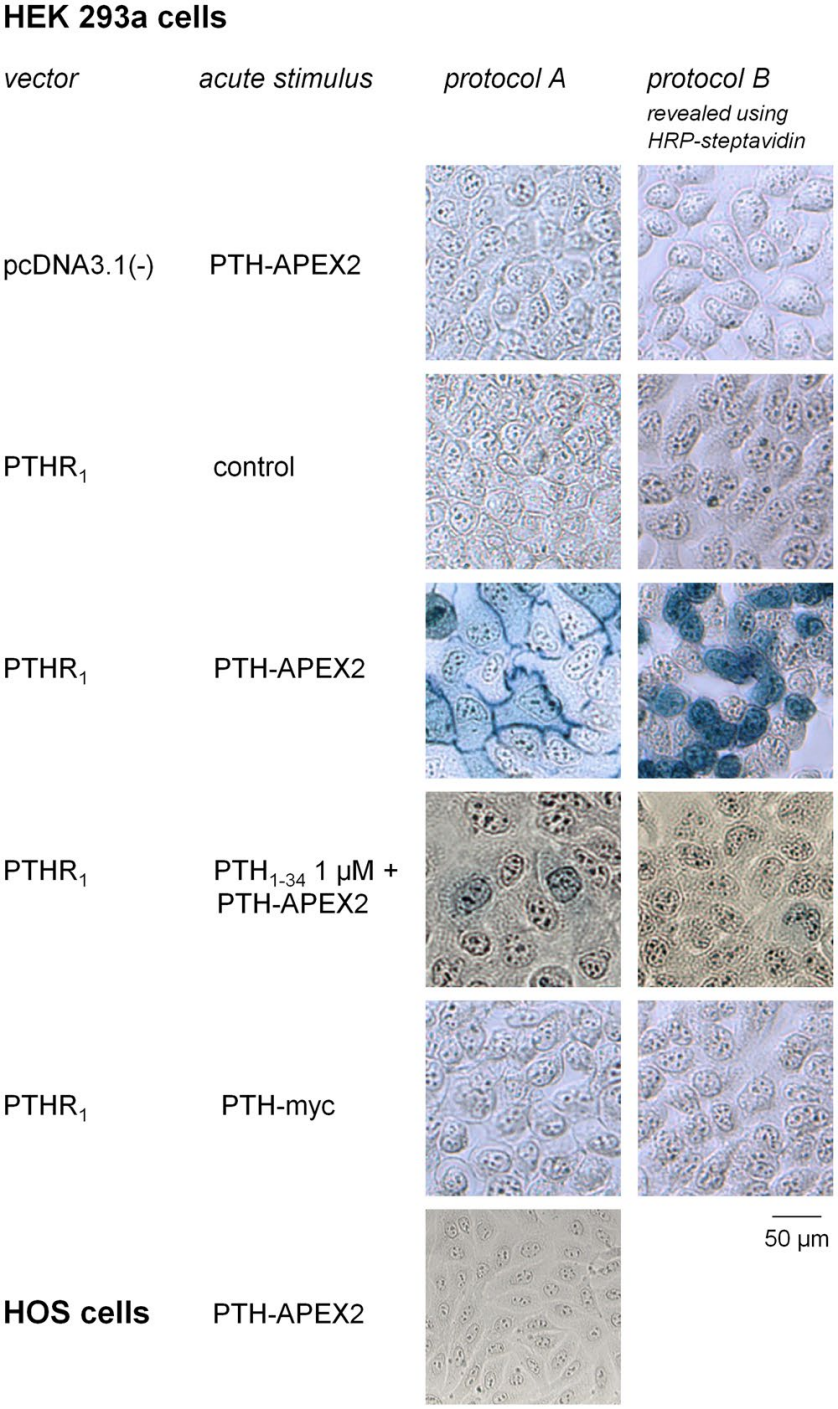
TrueBlue^TM^ labeling of PTHR_1_ expressing cells following stimulation with PTH-APEX2. Protocol A: HEK 293a cells transfected with either pcDNA3.1 or PTHR_1_ were stimulated for 30 minutes with PTH-APEX2 followed by a staining with TrueBlue^TM^. Optionally, cells were co-stimulated with PTH_1–34_ to evaluate the specificity of PTH-APEX2. The cells were observed in light transmission (final magnification 100×). Protocol B: a biotinyl-phenol signal amplification step followed incubation of cells with the receptor ligands, and a final incubation with streptavidin-HRP supported the staining with TrueBlue™, as outlined in Methods.

The second fusion protein, PTH-APEX2, possesses intrinsic peroxidase activity and was used without antibodies, a possible opportunity to improve the signal-to-noise ratio. PTH-APEX2 was applied to transfected HEK 293a cells, followed by a treatment with the TrueBlue™ solution (protocol A; Fig. 5). The HEK 293a cells expressing PTHR_1_, but not HOS cells, were strongly stained by the blue precipitate formed by the TrueBlue™ oxidation product generated by the receptor bound PTH-APEX2. The HEK 293a cells showed a strong membrane labeling consistent with the distribution of the PTHR_1_. The specificity of this labeling was confirmed by a co-treatment with PTH_1–34_ 1 µM which completely prevented the labeling of these cells. Mock transfected cells (empty pcDNA3.1) were also negative for TrueBlue™ staining, corroborating the specificity of PTH-APEX2. Also, cells transfected with PTHR_1_ and treated with control CM or PTH-myc CM alone were not labeled. PTH-APEX2 was unable to detect PTHR_1_s naturally expressed by HOS cells under these conditions. In “protocol B”, a biotinyl-phenol signal amplification step followed the incubation of cells with the receptor ligands. Then, a final incubation with streptavidin-HRP which supported the staining with TrueBlue™, as outlined in Methods, was performed. As can be expected from this technique which labels the vicinity of the PTH-APEX2 binding sites with multiple biotin-protein complexes, TrueBlue^TM^ staining was stronger in intensity, less localized at the subcellular level, but as specific as that of protocol A (Fig. 5).

**Figure 6:**
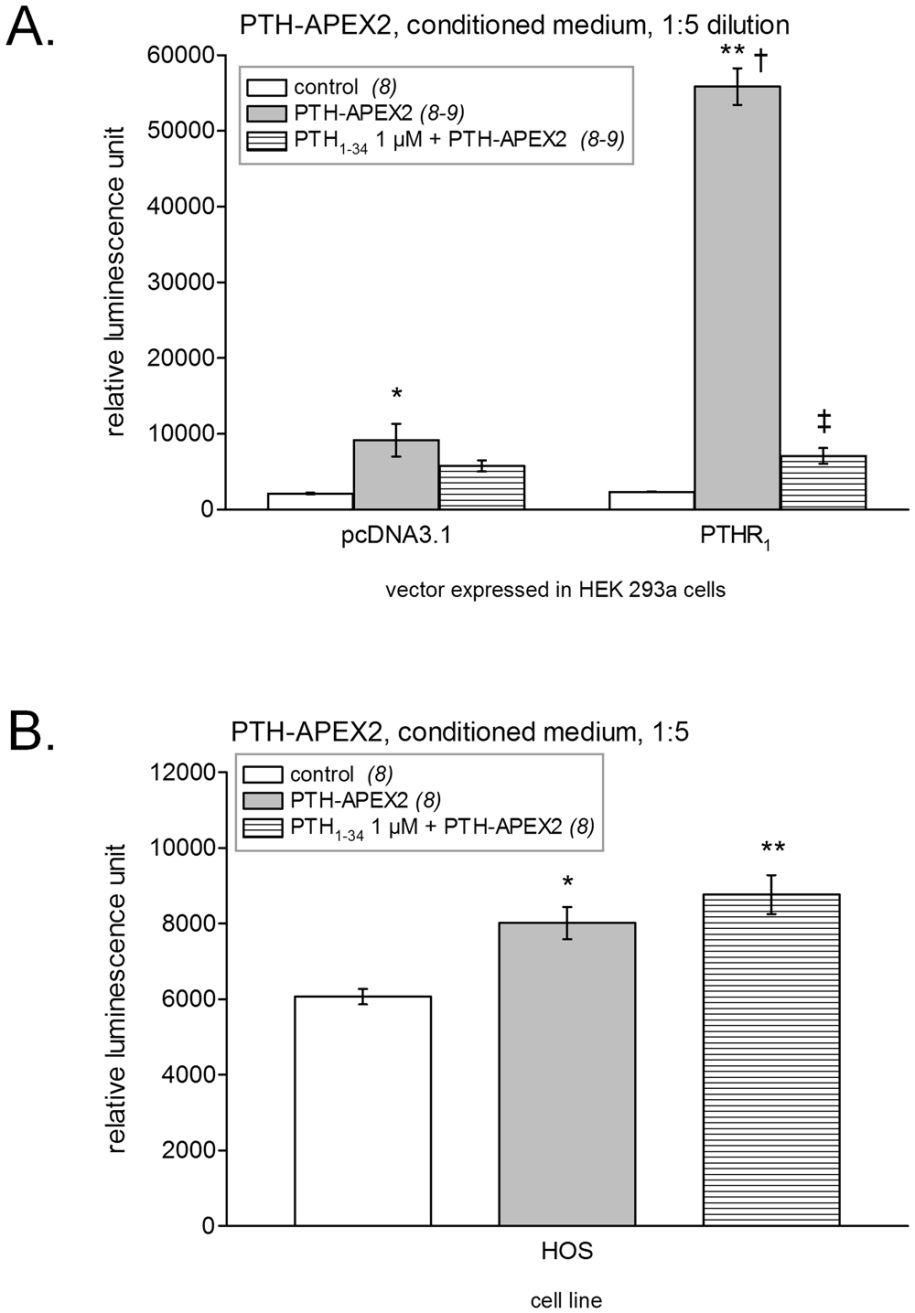
Luminescent detection of recombinant PTHR_1_s using the ligand PTH-APEX2. (**A**) Transfected HEK 293a (PTHR_1_ or pcDNA3.1) grown in opaque white 24-well plates were stimulated with PTH-APEX2 for 30 minutes at 37 °C. Following this incubation the cells were incubated with the Western Lightning®Plus ECL substrate allowing the luminescent detection of PTHR_1_ bound PTH-APEX2. ANOVA indicated that values were heterogenous (P < 10^−4^). Tukey’s multiple comparison test was applied to compare pairs of values. For each vector, values vs. their respective controls: *P < 0.05; **P < 0.001; effect of adding an excess of PTH_1–34_ vs. the enzymatic ligand alone: ^‡^P < 0.001. Comparison of each experimental condition between vectors: ^†^P < 0.001. (**B**) Endogenous receptors investigated in HOS cells. ANOVA indicated that values were heterogenous (P < 0.001). Each treatment vs. control value: *P < 0.01; **P < 0.001; effect of adding an excess of PTH_1–34_ vs. the enzymatic ligand alone: not significant (Tukey’s multiple comparison test).

The ability of PTH-APEX2 to detect the PTHR_1_ was then evaluated using the luminescent assay in which the receptor bound PTH-APEX2 produces light through the oxidation of luminol (Fig. 6A). The treatment of PTHR_1_-expressing HEK 293a cells with PTH-APEX2 (diluted 1:5 to reduce background noise) led to a strong signal (P < 0.001 vs. that of receptor-expressing control cells) that was significantly reduced in presence of PTH_1–34_ 1 µM (P < 0.001). The signal-to-noise ratio was much better than with the trimolecular PTH-myc complex, with a lower but still significant non-specific binding to mock-transfected cells (P < 0.05). However, the small but significant reaction of PTH-APEX2 with HOS cells (P < 0.05) was not abated by an excess of PTH_1–34_, thus failing to document specific binding to PTHR_1_s. (Fig. 6B).

### Labeling of PTHR_1_ expressing cells with PTH-HRP

**Figure 7:**
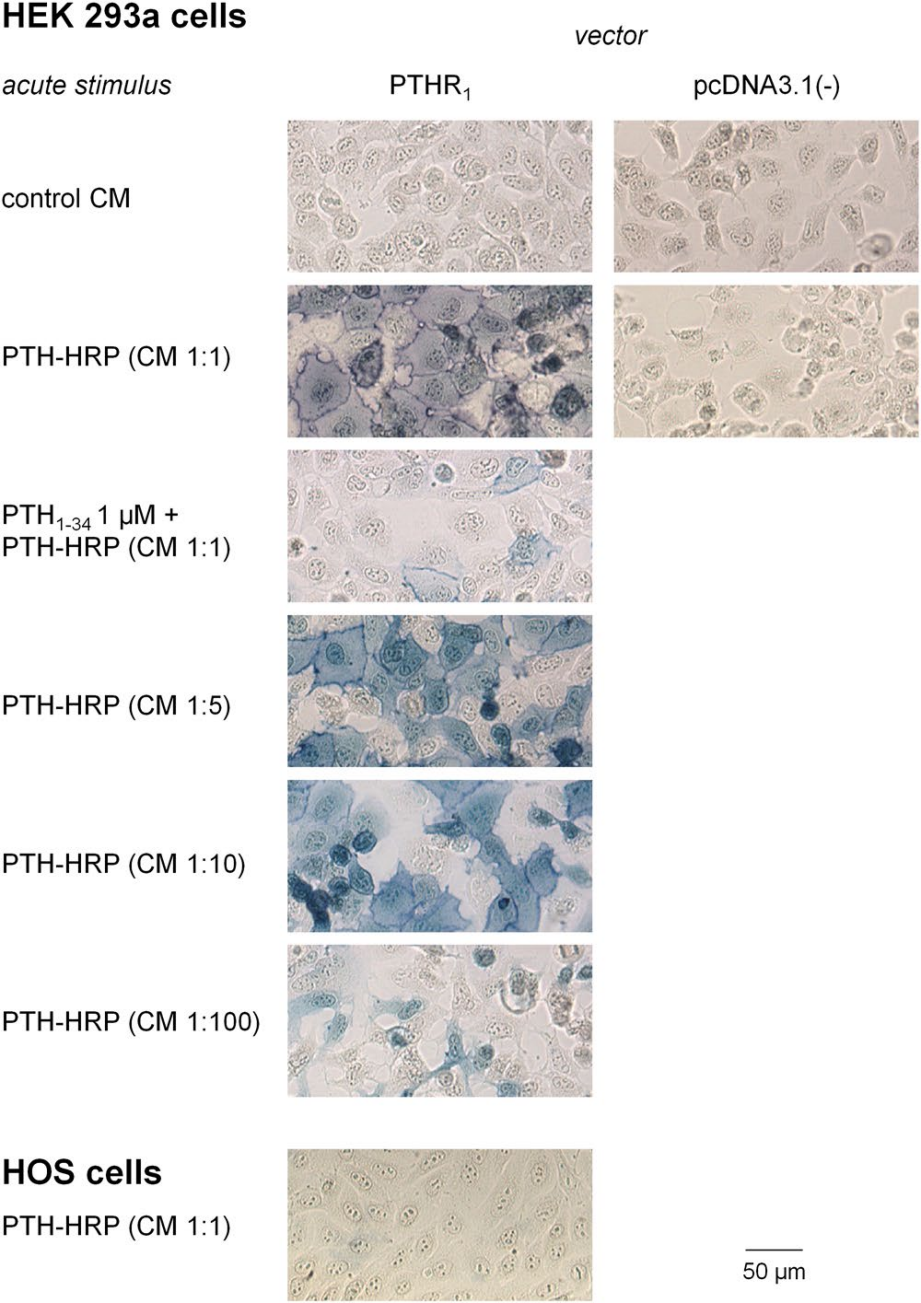
Detection of the PTH-HRP bound to recombinant (HEK 293a cells) or endogenous (HOS) PTHR_1_s using the colorimetric substrate TrueBlue™. Successive dilutions of PTH-HRP were used to evaluate the sensitivity of this fusion protein and the specificity of this fusion protein was confirmed by the effect of an excess of PTH_1–34_.

**Figure 8:**
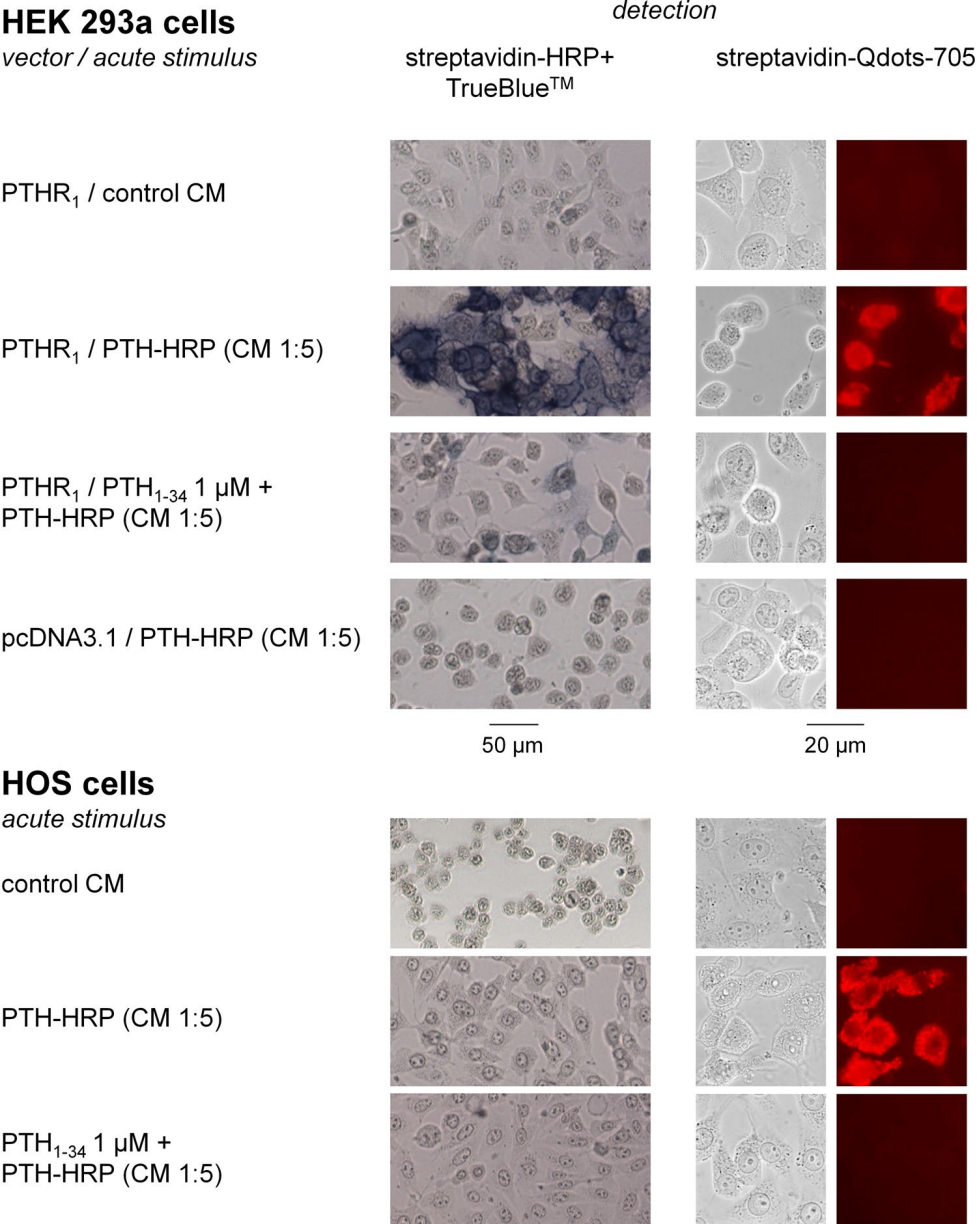
Amplified microscopic detection of PTHR_1_s using the ligand PTH-HRP. The primary reaction of this ligand was with biotin-phenol (“protocol B”); the secondary detection with either streptavidin-HRP + TrueBlue^TM^ or streptavidin-Qdots. These schemes were applied to signals generated by PTH-HRP either in transfected HEK 293a cells or in HOS cells. Transmission and epifluorescence, original magnification: 100 × (TrueBlue™) or 1000 × (Qdots).

The other enzymatic fusion protein that was designed and evaluated was PTH-HRP. The ability of PTH-HRP to detect recombinant populations of PTHR_1_s was first studied using the histochemical co-substrate TrueBlue™ (Fig. 7). The PTH-HRP fusion protein strongly labeled PTHR_1_ expressing cells, mostly at the plasma membrane levels, and the previously applied controls again supported specificity (co-treatment with PTH_1–34_ 1 µM, transfection with the empty vector). Undiluted PTH-HRP CM produced the strongest labeling of all three evaluated fusion proteins and was able to detect PTHR_1_ even when the CM was diluted up to 1:100 with fresh culture medium. However, PTH-HRP failed to detect endogenous populations of PTHR_1_s in HOS cells using the TrueBlue™ peroxidase substrate. Still, these experiments suggested that PTH-HRP was the best enzymatic fusion protein tested, and it was applied to “protocol B” amplification with biotin-phenol in transfected HEK 293a and HOS cells. The biotinylation of multiple cell surface proteins by this technique was confirmed by the detection of a complex fingerprint of streptavidin-HRP reactive bands in an immunoblot of the migrated proteins from HEK 293a cells expressing the receptor and treated with PTH-HRP, with or without excess PTH_1–34_, and biotin-phenol (Supplementary Fig. 1). The application of “protocol B” to PTH-HRP produced a very intense and specific staining of the recombinant receptor-expressing HEK 293a cells, but not of HOS cells (Fig. 8). However, the alternate detection of cell-bound biotin by streptavidin-Qdot 705 conjugates succeeded to detect receptors in a specific manner in both HEK 293a and HOS cells (Fig. 8; staining that appeared endosomal, abolished by a co-treatment with PTH_1–34_). Therefore, PTH-HRP supported the detection of endogenous populations of PTHR_1_ under specific experimental conditions.

**Figure 9:**
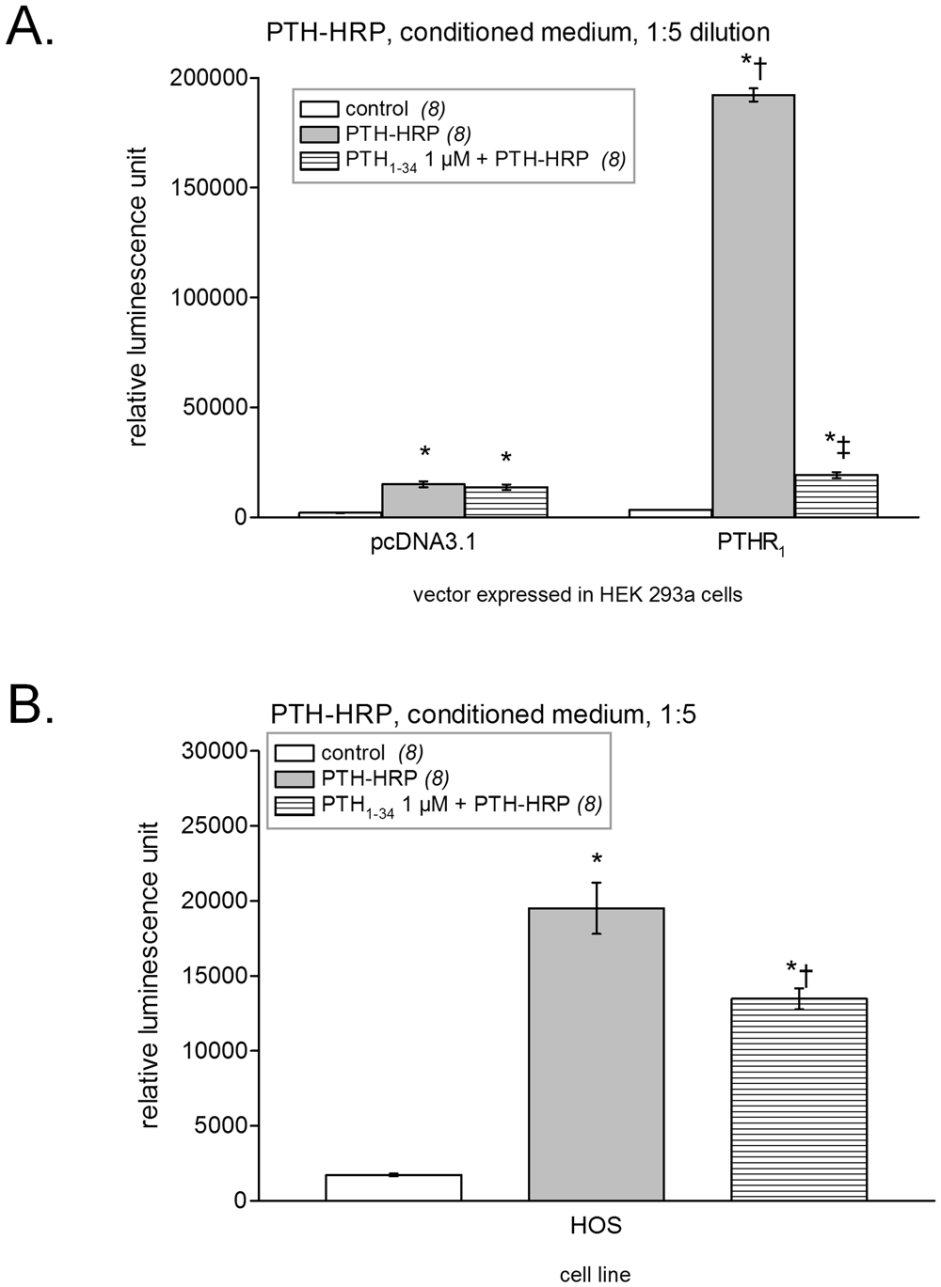
Luminescent detection of PTHR_1_s using the ligand PTH-HRP (CM diluted 1:5). (**A**) HEK 293a cells transfected with either an empty vector or one encoding for the PTHR_1_. The number of replicates is indicated between parentheses. ANOVA indicated that values were heterogenous (P < 10^−4^). Tukey’s multiple comparison test was applied to compare pairs of values. For each vector, values vs. their respective controls: ^*^P < 0.001; effect of adding an excess of PTH_1–34_ vs. the enzymatic ligand alone: ^‡^P < 0.001. Comparison of each experimental condition between vectors: ^†^P < 0.001. (**B**) Endogenous receptors detected in HOS cells. ANOVA indicated that values were heterogenous (P < 10^−4^). Each treatment vs. control value: ^*^P < 0.001; effect of adding an excess of PTH_1–34_ vs. the enzymatic ligand alone: ^†^P < 0.001 (Tukey’s multiple comparison test).

**Figure 10:**
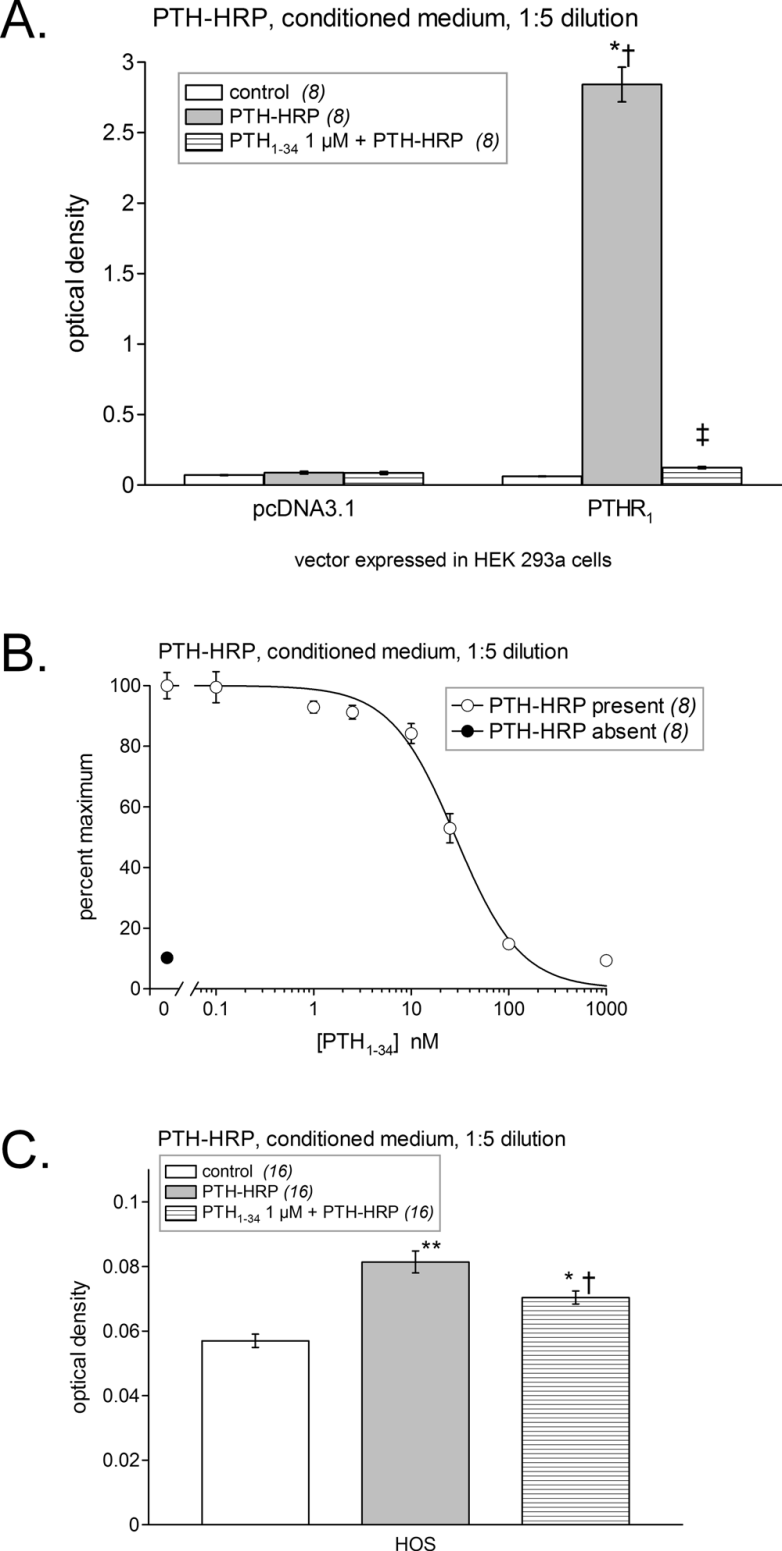
Alternate colorimetric detection of PTHR_1_s using the ligand PTH-HRP (CM diluted 1:5) and the TMB co-subtrate. (**A**) HEK 293a cells were transfected with an empty vector or that encoding PTHR_1_. The number of replicates is indicated between parentheses. ANOVA indicated that values were heterogenous (P < 10^−4^). Tukey’s multiple comparison test was applied to compare pairs of values. For each vector, values vs. their respective controls: ^*^P < 0.001; effect of adding an excess of PTH_1–34_ vs. the enzymatic ligand alone: ^‡^P < 0.001. Comparison of each experimental condition between vectors: ^†^P < 0.001. (**B**) Generation of a competition curve for PTH-HRP binding by varying concentrations of PTH_1–34_ in HEK 293a cells transfected with PTHR_1_ vector (results expressed in % of maximal optical density). (**C**) The TMB technique applied to HOS cells showed a significant signal (ANOVA P < 10^−4^; each treatment vs. control value: *P < 0.01; **P < 0.001; effect of adding an excess of PTH_1–34_ vs. the enzymatic ligand alone: ^†^P < 0.05, Tukey’s multiple comparison test).

We then validated the great potential of PTH-HRP using the luminescence assay (Fig. 9A). Diluted (1:5) PTH-HRP CM produced a very strong labeling of PTHR_1_ expressing cells (P < 0.001 vs. control receptor-expressing cells) while producing only a small but significant labeling of the mock-transfected cells (P < 0.001 vs. control mock-transfected cells). In fact, PTH-HRP produced the strongest luminescent signal of all three evaluated fusion protein (>4 fold) and also the best signal-to-noise ratio. Once again the addition of an excess of PTH_1–34_ significantly reduced the luminescent signal (P < 0.001) confirming the PTHR_1_ specificity of PTH-HRP. Using the luminescent assay, we were able to detect endogenous PTHR_1_s expressed at the surface of HOS cells using PTH-HRP. Indeed, this fusion protein produced a strong luminescent signal (P < 0.001) that was at least in part specific because the addition of PTH_1–34_ (1 μM) significantly reduced it (P < 0.001; Fig. 9B). An alternate detection protocol for PTHR_1_ expressed in HEK 293a grown in 24-well plates was based on the colorimetric peroxidase substrate TMB. In these experiments, PTH-HRP produced a very strong signal in receptor-expressing cells (P < 0.001) with no significant background. The labeling specificity was good, as judged from the effect of an excess PTH_1–34_ competing for PTH-HRP binding (P < 0.001; Fig. 10A). By varying the concentration of PTH_1–34_ in competition with a fixed dilution of PTH-HRP in cells that express recombinant PTHR_1_, an IC_50_ of 29 nM can be estimated (Fig. 10B). The K_I_ value of this peptide is reportedly 2 nM^11^, suggesting that PTH-HRP (used at 1.38 nM according to the enzyme immunoassay) has a high affinity for PTHR_1_. Further, PTH-HRP revealed by TMB supported the detection of PTHR_1_s in HOS cells in a statistically significant manner (P < 0.001) with a significant inhibitory effect of the PTH_1–34_ excess (P < 0.05; Fig. 10C). Thus, PTH-HRP enabled the detection of the small endogenous populations of PTHR_1_ using three distinct approaches: microscopy, luminescence and TMB colorimetry.

### Distribution of PTHR_1_ at the macroscopic scale

Plates of cell wells are macroscopic objects that emit bluish light when a suitable PTHR_1_ ligand, like PTH-APEX2, is revealed with the luminol co-substrate; this light is detected by placing the plate under a photographic film or in the reading chamber of the IVIS apparatus (Supplementary Fig. 2). The standard verifications of specificity are also applicable (signal abated by an excess of PTH_1–34_, absent in cells that do not express receptors).

## Discussion

Using a rational design strategy based on the literature and on our previous findings, two new fusion proteins with peroxidase activity were produced for the PTHR_1_. Indeed, these fusion proteins, PTH-HRP and PTH-APEX2, were evaluated along the previously reported PTH-myc construction^14^ for their ability to bind to recombinant and endogenous PTHR_1_s via intrinsic or assembled peroxidase domains. The expression and secretion of fusion proteins was evaluated in producer cells through immunoblot and an anti-PTH_1–84_ ELISA, showing accumulation of nanomolar concentrations of the mostly intact fusion proteins in the CMs. There seems to be some discrepancies in the results from both experiments because the concentrations determined in the PTH ELISA do not match the immunoblot signal densities, especially weak for PTH-myc. The exploited ELISA detects the C-terminal sequence of PTH probably leading to an underappreciation of the real quantity of PTH fusion proteins depending on the C-terminally fused sequence. Through their intrinsic peroxidase activity, PTH-APEX2 and PTH-HRP supported the detection of recombinant PTHR_1_s transiently expressed in HEK 293a using various detection protocols based on widely available substrates and co-substrates systems. The detection of PTHR_1_s in these cells was also possible with a co-stimulation of a trimolecular complex composed of PTH-myc, a mouse monoclonal anti-myc antibody and an anti-mouse HRP-conjugated antibody. A standard pharmacologic principle inspired from radioligand binding assays was systematically applied to recognize the fraction of the cell binding that is specific, i.e. that can be attributed to receptor binding. This specific fraction is the one that is supressed by a co-treatment with an excess of a known receptor ligand, PTH_1–34_ (1 μM). The use of mock-transfected HEK 293a cells provided an additional estimate of the non-specific binding of the fusion proteins to cells in both microscopic and quantitative enzymatic assays.

The three PTH fusion proteins do not perform equally well depending on the assays, and the assays themselves were not equally sensitive. In microscopy experiments, the TrueBlue^TM^ peroxidase co-substrate revealed only recombinant PTHR_1_s expressed at high density, but with excellent specificity (Figs 3, 5, 7, 8). TrueBlue^TM^ staining was negative in HOS cells treated with any of the 3 fusion proteins. PTH-myc is prone to a fairly large non-specific binding in the luminescence assay (Fig. 4), possibly due to the required co-treatment with 2 types of antibodies to confer enzymatic activity to it; in addition, it exhibits a lower potency than PTH-HRP in the cyclic AMP generation assay (Fig. 2C)^14^, consistent with the retained activity of PTH-HRP at high dilutions (Fig. 7). As for the enzymatic ligands, PTH-APEX2 is inferior to PTH-HRP for the intensity, signal-to-noise ratio and specificity of the luminol-based readouts (Figs 6 and 9). Further, PTH-APEX2 did not detect specific binding to endogenous PTHR_1_s in HOS cells. Perhaps the stronger signal of HRP vs. APEX2 derives from the fact that the latter is a peroxidase engineered to be functional in reducing conditions^17^. Also, APEX2 is much more susceptible than HRP to irreversible inhibition resulting from the enzyme activity. Indeed, in standard conditions, APEX2 can catalyse 3000–4000 reaction cycles before becoming permanently inactivated whereas HRP can catalyse more than 130,000 reaction cycles under the same conditions. This is probably due to the absence of a catalase activity in APEX2 that is present in HRP^17^. PTH-HRP was further tested in HOS cells, endogenously expressing the PTHR_1_^18^. Using the very bright fluorescent nanomaterial Qdots, PTH-HRP supported the detection of an endogenously expressed population of receptors in HOS cells. Other techniques based on luminescent and colorimetric substrates, detected this naturally expressed population of receptors following binding of PTH-HRP to HOS cells, albeit with a small specific signal. Nevertheless, PTH-HRP is a much more sensitive specific probe than the previously reported constructions PTH_1–34_-EGFP and PTH-myc^13,14^.

A promising application based on PTH-HRP is illustrated in Fig. 10B. PTH-HRP is adaptable to efficient, safe, cheap and reliable high throughput procedure for the screening of drugs in a binding competition assay, dispensing of an expensive and hazardous radioligand. Since, all class B GPCRs bind their receptor in the same fashion as PTH^19^, it is reasonable to assume that this fusion protein design strategy and arising applications could be applicable to other receptors of this family. Additional non-peroxidase enzymes for the design of ligands, such as the hydrolase β-galactosidase, may prove useful for certain applications and are supported as well by widely available detection reagents. Unlike peroxidases, such hydrolases are not “suicide” enzymes and may prove to be more flexible for accumulating signal over time. Finally, ligand-based fusion proteins could be generally less species-specific than antibodies across mammalian species barriers.

This work demonstrated the high potential of enzymatic fusion protein ligands of the PTHR_1_ notably to detect naturally expressed PTHR_1_s and perform high throughput screening for new ligands of this receptor.

## Methods

### Construction of the PTH-APEX2 plasmid

A genetically available mutant (K14D, W41F, E112K and A134P) of the APX soybean peroxidase called APEX2^17^ was fused to the C-terminus of the prepro-PTH_1-84_ sequence (Fig. 1). Secretion of PTH-APEX2 is predicted because the signal peptide and pro-peptide sequences of PTH were retained. From the pcDNA3-APEX2-NES vector (a gift from Dr. Alice Ting; plasmid # 49386, Addgene, Cambridge, MA, USA), the sequence corresponding to APEX2 was amplified by PCR using the following primers: 5′-AA TCC CAG GAC TAC AAG GAT GAC GAC G-3′ (sense) and 5′-TTG GTA CCG AGC TCG TTA GTC CAG GGT CAG GCG-3′ (antisense). Then, the sequence coding for the PTH domain was amplified from the PTH-myc vector (Origene Technologies, Rockville, MD, USA; cat # RC219848) using the following primers: 5′-C GTT TAA ACG GGC CCT ATG ATA CCT GCA AAA GAC ATG-3′ (sense) and 5′-CTT GTA GTC CTG GGA TTT AGC TTT AGT TAA TAC-3′(antisense). Using the Gibson assembly technique, both fragments were ligated in the XbaI/BamHI digestion product of the pcDNA3.1 vector to generate the PTH-APEX2 vector (Gibson Assembly Master Mix used as directed, New England Biology, Ipswich, MA, USA). The insert coding for PTH-APEX2 was validated by automated sequencing.

### Construction of the PTH-HRP plasmid

To generate PTH-HRP (Fig. 1), a vector coding for HRP was obtained (pCMV-erHRP, N175S mutant; a gift from Dr. Joshua Sanes; Addgene plasmid # 79909)^20^. The sequence coding for HRP was amplified using the following PCR primers: 5′-AA TCC CAG TAT CCA TAT GAT GTT CCA GAT TAT G-3′ (sense) and 5′-TTG GTA CCG AGC TCG TTA CAA GTC AGA GTT GCT GTT C-3′ (antisense). Then, the sequence coding for the prepro-PTH domain was amplified and assembled with the enzyme sequence precisely as for PTH-APEX2 from the vector PTH-myc using the following primers: 5′-C GTT TAA ACG GGC CCT ATG ATA CCT GCA AAA GAC-3′ (sense) and 5′-C ATA TGG ATA CTG GGA TTT AGC TTT AGT TAA TAC-3′ (antisense).

### Drugs and reagents

Cell culture reagents and buffers were purchased from Invitrogen/ThermoFisher (Waltham, MA, USA), PTH_1–34_ from R&D Systems (Minneapolis, MN, USA), Biotin-phenol from Iris Biotech GmbH (Marktredwitz, Germany), TrueBlue^TM^ from Kirkegaard & Perry Lab, Inc. (Gaithersburg, MD, USA). The 30% hydrogen peroxide solution and the 3,3′,5,5′-tetramethylbenzidine (TMB) solution were from Sigma-Aldrich (Oakville, ON, Canada) and the Western Lightning Plus-ECL substrate, from PerkinElmer (Woodbridge, ON, Canada).

### Cell culture and production of the fusion proteins

A subclone of HEK 293 cells, called HEK 293a, originally obtained from Sigma-Aldrich was used in most experiments. This cell type was grown in Dulbecco’s modified Eagle’s medium supplemented with 10% fetal bovine serum (FBS), 1% L-glutamine and 1% penicillin-streptomycin stock solutions (100×) (supplies from Invitrogen). The human osteosarcoma cell line HOS (originally obtained from the ATCC, Manassas, VA, USA; #CRL-1543) was chosen because it has been shown to express a functional, endogenous population of PTHR_1_s^18^. These cells were grown in α-Modified Eagle medium (Invitrogen) supplemented with 10% FBS, 1% L-glutamine and 1% penicillin-streptomycin stock solutions (100×). The cells were passaged twice a week.

Recipient HEK 293a cells were transfected with either the pcDNA3.1 empty vector (Invitrogen) or one encoding PTHR_1_ (vector was a generous gift from Dr. T. J. Gardella, Massachusetts General Hospital, Boston, USA). Briefly, 70% confluent cells were transected with polyethylenimine (PEI; Sigma-Aldrich) using a ratio of 3:1 (3 µg of PEI for each µg of DNA). The DNA was first diluted in OptiMEM (Invitrogen) before adding PEI and the mixture was left to incubate at room temperature for 30 minutes before adding it to the cells to be transfected. The next day, the medium of the transfected cell was discarded and replaced with fresh complete culture medium to allow them to grow. Forty-eight hours following transfection, the cells were used in various experiments.

The fusion proteins were produced as a conditioned medium (CM) by transfecting 70% confluent HEK 293a producer cells using PEI as described above. However, since the fusion proteins were secreted, the medium was not changed the day after the transfection. Seventy-two hours following transfection, the CM of transfected cells was collected from the flask and then centrifuged 10 minutes at 3500 rpm to remove cellular debris. Following centrifugation, the CM was aliquoted and stored at −20 °C. Cells were either transfected with one of the two previously described vectors or with a vector coding for the previously reported construction PTH-myc^14^. This vector, myc- and DDK-tagged pro-PTH, was purchased from Oriene Technologies (catalog number RC519848) and encodes prepro-PTH_1-84_ peptide extended at its C-terminus with two epitopes in tandem, myc and DDK. The CMs were quantified using an ELISA for intact PTH (Genway Biotech Inc., San Diego, CA, USA).

The 3 types of fusion proteins have been used in binding experiments to cells that express no receptor or recombinant or naturally expressed PTHR_1_s_;_ the 30 min incubation period at 37 °C was followed by extensive rinsing and various protocols for the enzymatic detection of the receptor-bound ligand, as specified below. The non-specific binding was defined as the fraction of the ligand-mediated signal that was resistant to competition when the recipient cells were co-treated with an excess of the specific agonist PTH_1–34_ (1 μM), as it is customary in radioligand binding assays.

### Immunoblots

The identity of secreted fusion proteins was verified in immunoblotting experiments (10 μl of CM per track on 15% polyacrylamide gels) performed as previously described^21^ using various antibodies. We used the anti-myc tag monoclonal antibody (clone 4A6, EMD Millipore, ON, Canada) and the HRP-conjugated anti-PTH antibody provided with the anti-PTH ELISA kit mentioned above. A secondary anti-mouse antibody conjugated to HRP (Santa Cruz Biotechnologies, Dallas, TX, USA) was used to detect the presence of the anti-myc tag antibody. The FLAG tag was present in each construction but we were unable to detect this epitope perhaps due to the post-translational sulfatation of this epitope leading to poor recognition by the specific antibody^22^.

### Signaling induced by fusion proteins

The agonist action of PTH-related agonists was investigated using the expression of the transcription factor c-Fos, a distal signaling response to the stimulation of various receptor-ligand systems^14^. HEK 293a cells, expressing or not PTHR_1_, were stimulated for 1 hr with PTH_1–34_ or one of the CMs containing PTH fusion proteins. Total HEK 293a cell extracts were immunoblotted to detect c-Fos expression using the K-25 rabbit polyclonal antibodies (Santa Cruz Biotechnology; dilution 1:50 000). A commercial cyclic AMP ELISA kit (Cell Biolabs, San Diego, CA, USA) was applied as directed without the optional acylation reaction to quantify the intracellular second messenger of PTHR_1_-expressing HEK 293a cells (confluent 35-mm petri dishes) variously stimulated for 10 min (extract volume 600 μl/dish).

### TrueBlue™ histochemistry of peroxidase-labeled fusion proteins

For microscopic experiments involving the recombinant PTHR_1_, recipient HEK 293a cells were cultivated in 8 cm^2^ petri dishes before being transfected with an empty vector or a vector coding for PTHR_1_. Forty-eight hours following transfection, these receptor cells were stimulated with CMs from producer cells that contained an enzymatic or tagged PTHR_1_ ligand (30 minutes, 37 °C). After this incubation, these cells were washed 3 times with Hank’s balanced salt solution (HBSS) (Invitrogen) before adding 1 ml of TrueBlue^TM^ peroxidase substrate. The reaction was stopped when optimal coloration was reached by removing the TrueBlue^TM^ substrate and by washing with H_2_O. The cells were then observed using an Olympus BX51 microscope (Olympus, Richmond Hill, ON, Canada) coupled to a CoolSnap HQ digital camera (Photometrics, Tucson, AZ, USA) or a color camera (QImaging MicroPublisher 3.3RTV, QImaging, Surrey, ON, Canada) (final magnification 100×). HOS cells expressing an endogenous population of PTH_1_ receptors were treated using a similar protocol.

### Biotin-phenol or biotinyl-tyramide detection of peroxidase-labeled fusion proteins

A two-step process for the detection of GPCR bound fusion proteins was optionally applied to increase sensitivity. Following stimulation with the appropriate CM as described above, cells were rinsed 3 times with HBSS before being incubated with 100 mM H_2_O_2_ and biotinyl-tyramide (from the Tyramide Signal Amplification kit, ThermoFisher, used as directed) or 500 µM biotin-phenol in the TSA amplification buffer from the same kit for 15 minutes at room temperature. The GPCR-bound peroxidase catalysed the oxidation of biotin-phenol generating a very short-lived biotinphenoxyl radical which covalently tagged proteins proximal to the peroxidase. Following the oxidation of biotin-phenol, cells were rinsed 3 times with HBSS. Then, the cells were incubated 30 minutes with a HBSS solution containing 1% BSA to block the non-specific binding sites before being incubated with a horse radish peroxidase conjugate of streptavidin (SA-HRP; Invitrogen) for 1 hour. Finally, the cells were again rinsed 3 times with HBSS before the detection of the covalently bound biotin using TrueBlue™. An alternate protocol was also applied: SA-HRP was replaced by SA-Qdots-705 (dilution 1:200, ThermoFisher) and photographed as described^23^.

### Chemiluminescent and colorimetric detection of peroxidase-labeled fusion proteins

HEK 293a cells transfected with the appropriate vector (either pcDNA3.1 or PTHR_1_) or HOS cells were grown in white 24-well microplates with clear bottom (VisiPlate-24, PerkinElmer, Whaltham, MA, USA). Cells were treated with the CMs for 30 minutes at 37 °C followed by three rinsing with HBSS. After this step, 500 µL of the luminol-based Western Lightning Plus-ECL substrate (PerkinElmer) was added to each well. Using a TECAN Infinite® 200 PRO microplate reader (Tecan Trading AG, Männedorf, Switzerland), luminescence readings were obtained for each well. The colorimetric detection of PTHR_1_ bound fusion proteins was performed using TMB in clear 24-well microplates. Briefly, following the incubation with the fusion protein, cells were rinsed three times using HBSS and were further incubated 30 minutes at room temperature with 250 µL of a TMB solution. The reaction was stopped using an equivalent volume of 0.2 M HCl solution turning the blue oxidation product of TMB yellow, and the plate was read for absorbance at 450 nm using a TECAN Infinite® 200 PRO microplate reader.

### Data analysis

Numerical values are reported as mean ± s.e.m. Sets of values were compared using ANOVA followed by Tukey-Kramer multiple comparison test (GraphPad Prism computer program version 5.0; GraphPad, San Diego, CA).

## Electronic supplementary material

Supplementary Information
