# Supplementary material for: Production and evaluation of parathyroid hormone receptor1 ligands with intrinsic or assembled peroxidase domains
Source: Sci Rep. 2017 Oct 12;7:13099. doi: 10.1038/s41598-017-13548-0 (PMC5638942; doi:10.1038/s41598-017-13548-0)

Production and evaluation of parathyroid hormone $receptor_1$ ligands with intrinsic or assembled peroxidase domains

Xavier Charest-Morin, Patrice E. Poubelle, François Marceau

Supplementary methods

Cell protein biotinylation by the PTH-HRP biotin-phenol combination

In order to confirm the basic tenets of protein cell biotinylation, HEK 293a cells transfected with either pcDNA3.1 or $PTHR_1$ were stimulated with PTH-HRP, with an optional excess of PTH_{1-34} competitor, before being incubated with biotin-phenol and H_2O_2 for a period of 15 minutes (as for cell staining). Following this incubation, the cells were lysed and the total cell extract were migrated in a 9% gel and transferred to detect biotinylated proteins using streptavidin-HRP (dilution 1:5000).

Imaging of luminescence in macroscopic objects

The integrated imaging station IVIS Lumina LT Series III (PerkinElmer), along with its Living Image software, was exploited to evaluate the distribution of $PTHR_1$ in macroscopic objects using the luminescence generated by enzymatic ligands (Western Lightning Plus-ECL substrate as above). The apparatus was set in luminescence mode without filters, medium binning and automatic exposure. This experiment exploited HEK 293a cells grown in 24-well plates that transiently expressed $PTHR_1$ or pcDNA3.1. In addition, a photographic film commonly used for immunoblots was positioned over a 24-well plate containing the cells and the luminescence substrate.

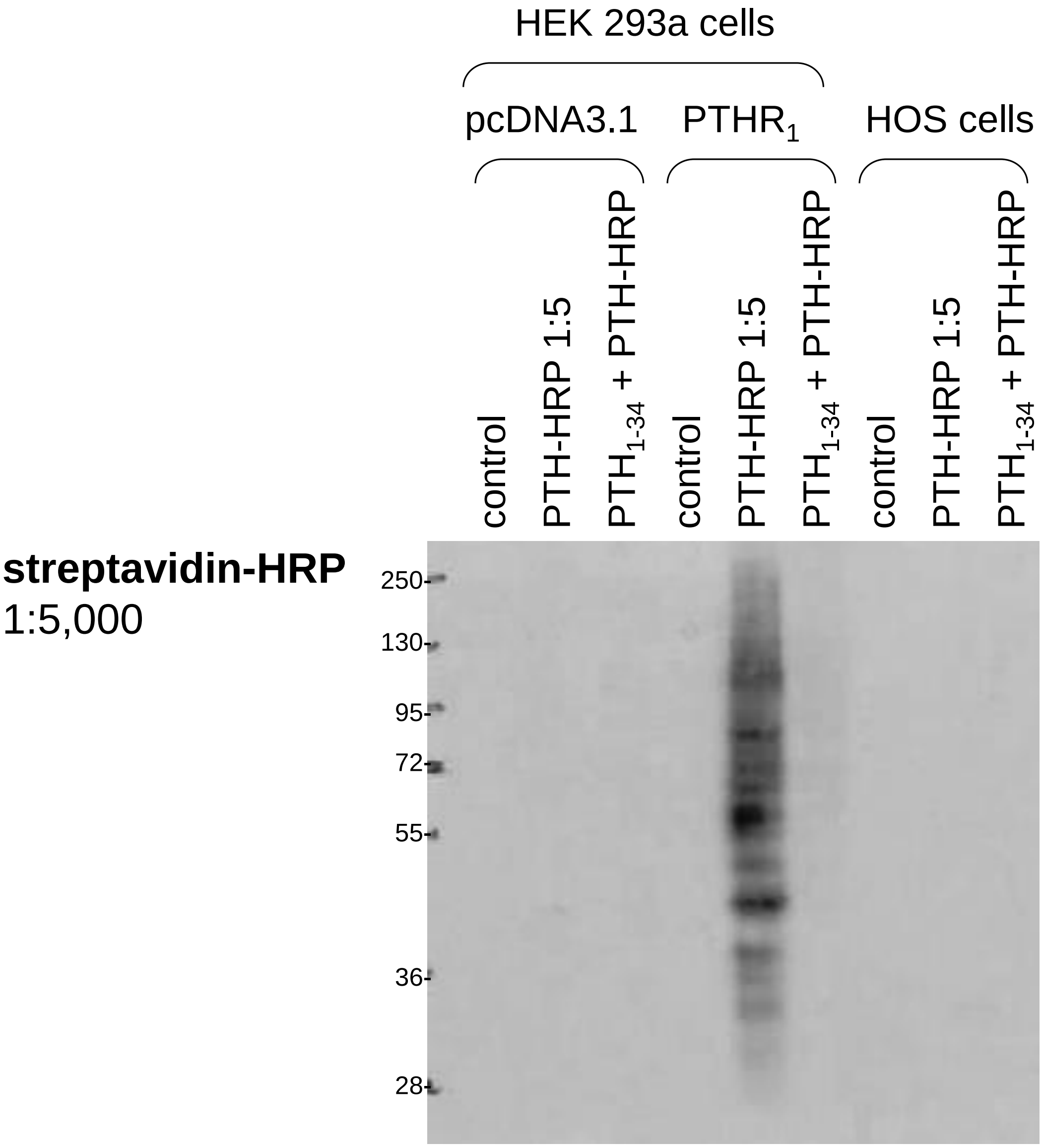

Supplementary Fig. 1. Blot of migrated extracts of HEK 293a or HOS cells submitted to successive treatments with PTH-HRP and the biotin-phenol co-substrate. A diffuse and specific fingerprint reactive with streptavidin-conjugated HRP is seen in the extract of HEK 293a cells expressing recombinant $PTHR_1$s, but not in HOS cells.

A.

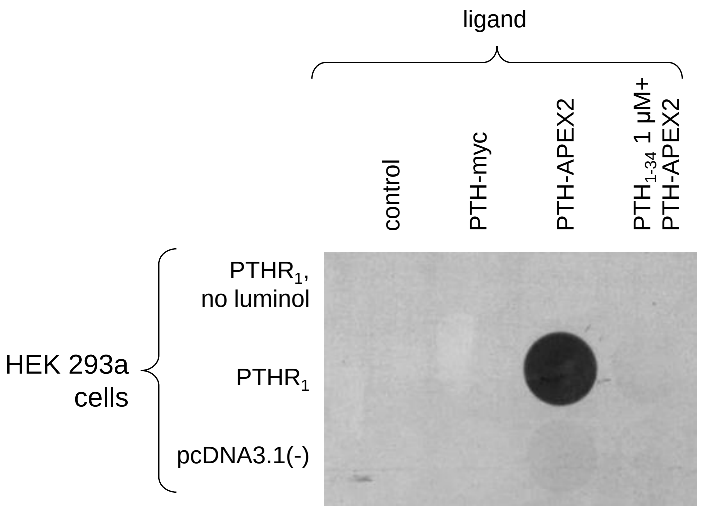

B.

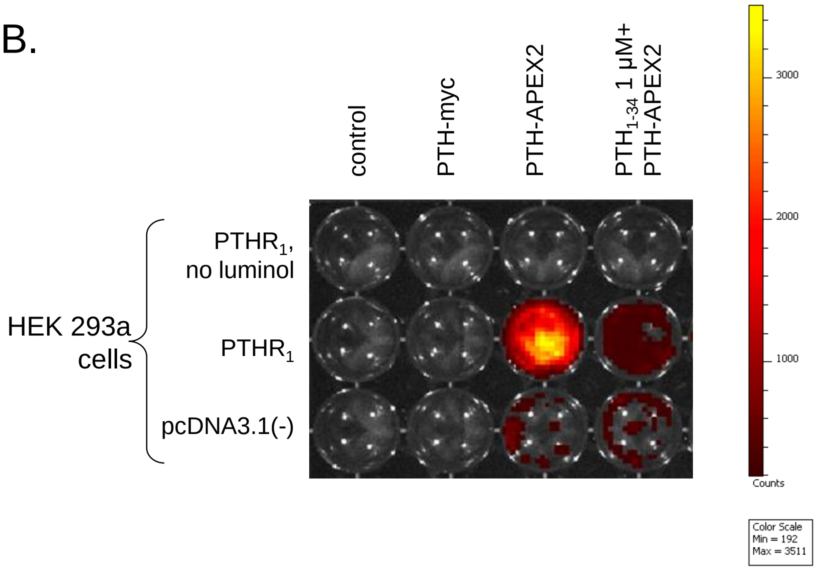

Supplementary Fig. 2. Optical detection of recombinant $PTHR_1$s expressed in HEK 293a cells in 12-well plates using an enzymatic ligand construction revealed by the luminol-based luminescent reagent. A. Photographic film reacts to the enzymatic construction PTH-APEX2 if $PTHR_1$ is expressed and if the luminol-based reagent is supplied, but not if an excess of PTH_{1-34} is added. The non-enzymatic ligand PTH-myc has no intrinsic effect. Representative of multiple experiments. B. Luminescence evaluated by the IVIS Lumina 3 apparatus. The activity of PTH-APEX2 is largely specific, being abated by competition with PTH_{1-34}. The signal disappears when luminol is not supplied. Representative of 2 experiments.

Full-length blot for Fig. 2A, top left

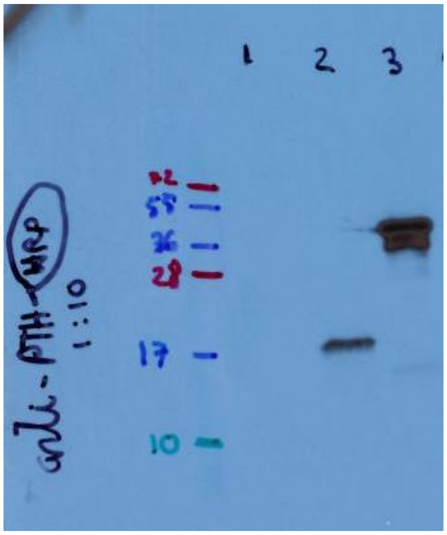

Full-length blot for Fig. 2A, top right

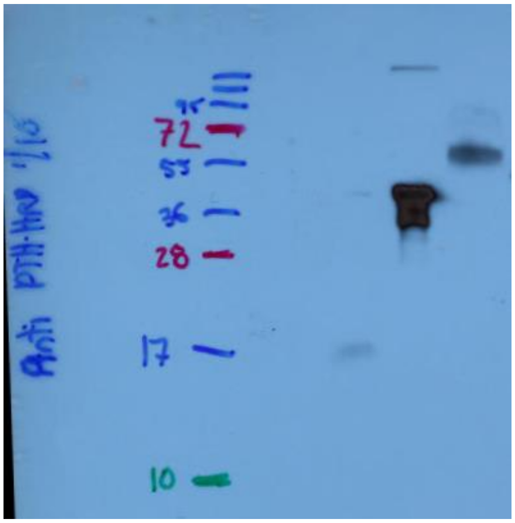

Full-length blot for Fig. 2A, top bottom

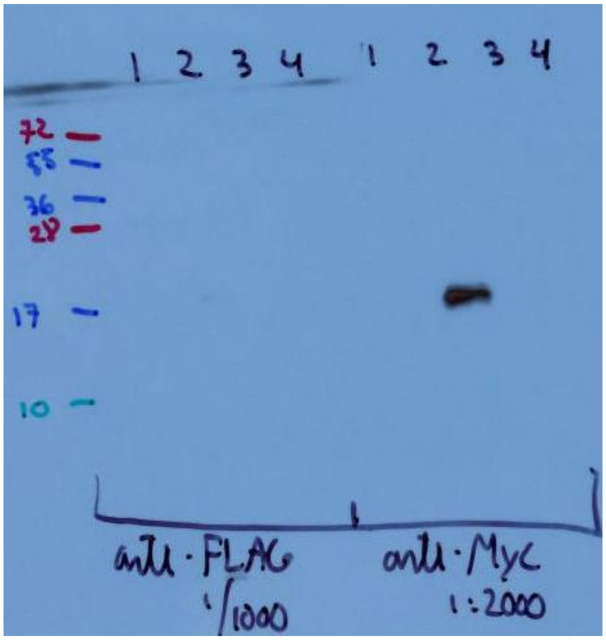

Full-length blot for Fig. 2B, top

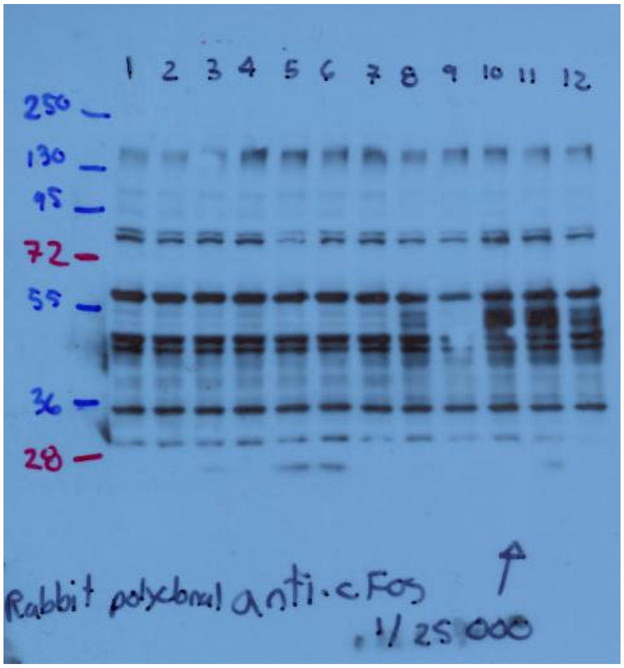

Full-length blot for Fig. 2B, bottom

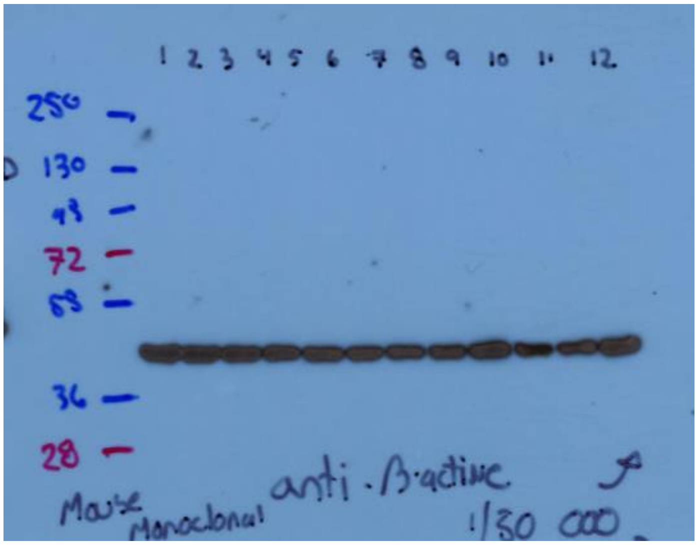

Full-length blot for Suppl. Fig. 1

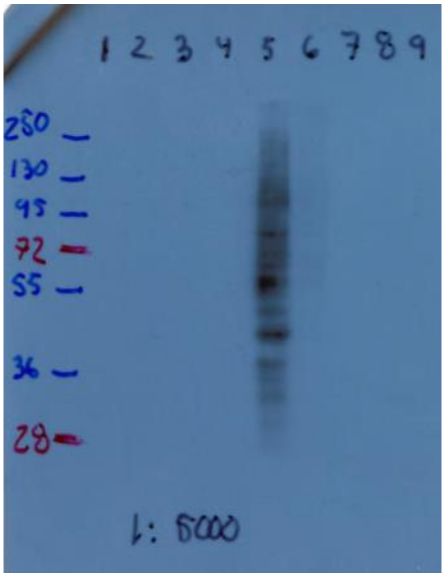

Supplement: Supplementary file 1 — Supplementary Information [file 41598_2017_13548_MOESM1_ESM.pdf]
